# Tumor necrosis factor alpha – an useful biomarker in a combined predictive model for liver fibrosis staging in patients with chronic HCV infection

**DOI:** 10.1186/1471-2334-14-S7-O16

**Published:** 2014-10-15

**Authors:** Daniela-Ioana Munteanu, Raluca Mihăilescu, Mihaela Andreea Rădulescu, Anca-Ruxandra Negru, Cătălin Tilişcan, Viorica Poghirc, Victoria Aramă, Ștefan Sorin Aramă

**Affiliations:** 1 National Institute for Infectious Diseases "Prof. Dr. Matei Balş", Bucharest, Romania; 2 Carol Davila University of Medicine and Pharmacy, Bucharest, Romania

## Background

Staging liver fibrosis in chronic HCV infection represents an important step for an individualized management. In the last decade the liver biopsy was less used for fibrosis staging due to its invasive nature and risk of complications. Multiple non-invasive methods were developed for the evaluation of liver fibrosis, none of these being an ideal one. The aim of this study was to evaluate the diagnostic accuracy of a new non-invasive method designed to differentiate patients with significant liver fibrosis from those without. (F2-F4 vs. F0-F1).

## Methods

We conducted a cross-sectional study in patients with chronic HCV infection, in a tertiary-care hospital, from November 2012 until April 2013. Blood samples were collected for: aspartat-aminotransferase (AST), alanin-aminotransferase (ALT), gamma-glutamyl transpeptidase (GGT), total bilirubin (TB), albumin, total cholesterol (CH), triglycerides (TG), fasting glucose (GLU), white blood cells (WBC), platelets (PLT) and tumor necrosis factor alpha (TNFa). We used DIAsource ImmunoAssay, Louvain-la-Neuve, Belgium, for TNFa plasma levels quantification. Liver fibrosis was estimated in all patients using Fibromax® (Biopredictive, France).

## Results

We included 114 consecutive patients and we divided them into 2 groups: estimation group – 79 patients and validation group – 35 patients. There were no significant differences between the 2 groups regarding sex ratio, median age, liver fibrosis score or biochemical and inflammation variables. We found a statistical correlation between the liver fibrosis score estimated by Fibromax and age, AST, ALT, GGT, TB, CH, GLU, PLT and TNFa. We constructed a regression model and subsequently a score combining age, TB, PLT count and TNFa that proved to be useful for identification of patients with significant liver fibrosis. The area under the ROC curve was 0.887 for the estimation group and 0.875 for the validation group. Using the best cut-off value (<1.06) the score positive predictive value was 90%. In the estimation group 51% (40/79) of patients were diagnosed with significant fibrosis whereas in the validation group the percent of patients with significant fibrosis was 54% (19/35).

## Conclusion

A score combining age of the patient, TB, PLT count and TNFa value could be an accessible and accurate tool for the identification of significant liver fibrosis in patients with chronic HCV infection.

